# Letter to the Editor: Difference between alveolar echinococcosis and hydatid disease

**DOI:** 10.1016/j.rmcr.2023.101967

**Published:** 2024-01-03

**Authors:** Gaëtan-Romain Joliat, Nermin Halkic

**Affiliations:** Department of Visceral Surgery, Lausanne University Hospital CHUV, University of Lausanne (UNIL), Lausanne, Switzerland

Dear Editor,

We read with interest the case report entitled “Non-resectable pulmonary alveolar echinococcosis with multi-stage vertebral location”, recently published in your journal [1]. While this article reports an interesting case of hydatid disease of peculiar location, we think that some important points need to be clarified.

First of all, we would like to underline that alveolar echinococcosis is not equal to hydatid disease. Alveolar echinococcosis is due to an infection by the tapeworm *Echinococcus multilocularis*, while hydatid disease (also known as hydatidosis or cystic echinococcosis) is caused by *Echinococcus granulosus*. These two types of echinococcosis have a completely different pathogenesis and clinical presentation. Alveolar echinococcosis can be compared to a malignant tumor with its propensity to infiltrate the liver parenchyma and to disseminate to distant organs. Hydatid disease, on the contrary, consists of parasitic cysts that develop in the liver and potentially in other organs, but without infiltrating the infected organ. In the abovementioned case report, the confusion already starts in the title and in the first sentence of the introduction, where alveolar echinococcosis is mentioned to be equivalent to hydatid cyst or hydatidosis, and then throughout the manuscript.

Geographical distribution of both diseases is also different. Alveolar echinococcosis is only present in the North Hemisphere and more specifically in endemic regions (e.g., China or continental Europe) [2]. Patients from non-endemic regions (such as North Africa) get infected if they have stayed in endemic countries. On the contrary, hydatid disease is cosmopolitan, but its incidence varies from a country to another [3].

Serology tests are important diagnostic tools. Practitioners use these tests as part of the etiological investigation of a cystic lesion of the liver or the lungs. ELISA or indirect hemagglutination tests using an antigen of *Echinococcus granulosus* do not permit to make the distinction between alveolar and cystic echinococcosis [2,4]*.* Other specific ELISA tests using antigens such as Em2 and reEm18 enable to clarify the diagnosis [2]. Unfortunately, the serological tests performed were not specified in the present case report. However, clinical and radiological presentation confirmed it was a hydatid cyst.

We wanted to highlight these specific points because we think that it is important to distinguish alveolar echinococcosis from hydatid disease, as these two pathologies are completely different with dissimilar pathogenicity, treatments, and prognoses.

## Financial support

None.

## Declaration of competing interest

None.
